# Phenolic Extracts from Wild Olive Leaves and Their Potential as Edible Oils Antioxidants

**DOI:** 10.3390/foods2010018

**Published:** 2013-01-04

**Authors:** Theodora-Ioanna Lafka, Andriana E. Lazou, Vassilia J. Sinanoglou, Evangelos S. Lazos

**Affiliations:** 1 Laboratory of Food Processing, Department of Food Technology, Technological Educational Institute of Athens, Agiou Spyridonos St., 12210, Egaleo, Athens, Greece; E-Mails: theodoralafka@hotmail.com (T.-I.L.); v_sinanoglou@yahoo.gr (V.J.S.); 2 Laboratory of Process Analysis and Design, School of Chemical Engineering, National Technical University of Athens, Zografou Campus, 15780, Athens, Greece; E-Mail: alazou@central.ntua.gr

**Keywords:** antioxidants, extraction kinetics, oil protection, phenolics, supercritical fluid extraction

## Abstract

The kinetics solid-liquid extraction of phenolics from wild olive leaves was elaborated using different mathematical models (Peleg, second order, Elovich, and power law model). As solvents, methanol, ethanol, ethanol:water 1:1, *n*-propanol, isopropanol and ethyl acetate were used. The second order model best described the solvent extraction process, followed by the Elovich model. The most effective solvent was ethanol with optimum phenol extraction conditions 180 min, solvent to sample ratio 5:1 v/w and pH 2. Ethanol extract exhibited the highest antiradical activity among solvent and supercritical fluid extraction (SFE) extracts, which in addition showed the highest antioxidant capacity compared to synthetic and natural food antioxidants such as BHT, ascorbyl palmitate and vitamin E. Antioxidant potential of SFE extract was quite high, although its phenolic potential was not. Leaf extracts were proven to be good protectors for olive and sunflower oils at levels of 150 ppm.

## 1. Introduction

Olive mill and olive processing residues are attractive sources of natural antioxidants. An important part of these residues is olive tree leaves (usually 5%, but possibly reaching up to 10% of the total olives’ weight depending on practices applied). In addition, during olive tree cultivation, the pruning step generates a considerable volume of olive leaves, which are usually used as animal feed, and which could also be used for antioxidant or olive-leaf extract production [1,2,3,4,5]. Olive leaves themselves have been used as a remedy against various diseases, while olive-leaf extract has been reported to have antioxidant capacity, antimicrobial activity, anti-HIV properties, vasodilator effect, and hypoglycaemic effect [1,6]. In the past few years, the demand for olive leaf extract has increased for use in foodstuffs, food additives and functional food materials. Although the antioxidant activities of some single phenolic compounds in olive leaf are well known, antioxidant activities of its extract from various solvents, and especially from wild olive varieties for which higher phenolics content is expected [7], have not been clearly investigated.

As each plant material has its unique properties in terms of phenolic extraction, it is very important to develop the optimal extraction conditions and afterwards the extract evaluation in terms of antioxidant activity and composition, as well as further utilization. Solvent extraction is a process designed to separate soluble phenolic compounds by diffusion from olive leaves (solid matrix) using a solvent (liquid matrix). Many factors contribute to the efficiency of the solvent extraction process, such as solvent type, pH, temperature, the number of extraction steps, solvent/solid ratio, and particle size of the solid matrix [8,9,10]. Furthermore, supercritical fluid extraction (SFE) can be used to obtain various bioactive compounds without any solvent residue and safety hazard. CO_2_-SFE is the most favored method for phenolic compounds isolation, which leads to higher phenol recoveries than sonication in liquid solvents. SFE extraction yield was low (only 45%), using liquid methanol [11]. More recently, during oleuropein extraction from olive leaves, the use of CO_2_-SFE alone had been characterized as not satisfactory, needing a polar modifier to improve yield and selectivity of the process [12].

Considering that the extractability depends mainly on solvent type and on extraction method, and that it is difficult to recommend a suitable extracting solvent for individual plant materials, due to variation of bioactives in different plant materials, the present investigation was undertaken to study and determine phenols extraction conditions from the wild olive tree leaves using various solvents, as well as to evaluate and compare the phenolic content and the antioxidant activity of solvent and SFE phenolic extracts and best extract application to oil protection.

## 2. Experimental Section

### 2.1. Samples and Reagents

Olive leaves were collected from wild olive trees, variety Agrielia (*Olea europaea* var. *oleaster* (Hoffm. & Link.), Synonyme: *Olea europaea* var. *sylvestris* (Mill.)), from Attica area, Greece. The olive leaves were analyzed for moisture, total solids, fat, and ash according to AOAC [13] methods.

Analytical grade methanol, ethanol, *n*-propanol, isopropanol and ethyl acetate, as well as HPLC grade acetonitrile, acetic acid and water were purchased from Merck (Darmstadt, Germany). Folin-Ciocalteau phenol reagent and 2,2-diphenyl-1-picryl-hydrazyl (DPPH) were purchased from Sigma Chemical Co. (Sigma-Aldrich Company Ltd., Dorset, Great Britain), while SFC-grade carbon dioxide was purchased from Air Liquide (Paris, France).

### 2.2. Extraction of Phenolic Compounds

#### 2.2.1. Solvent Extraction

Olive leaves were ground using a Brabender mill to pass through a 0.500 mm sieve. Based on previous findings [4,10] concerning the effects of extraction parameters on phenolics extraction, and taking into account that high temperatures, though causing an increase in total phenolics obtained, definitely harm antioxidant compounds, extraction was performed at room temperature (25 °C), while the pH was adjusted to 2.0. The latter was based on the fact that acidification of olive leaves increases phenolic compounds’ solubility and diffusion from the plant material through cell wall disintegration, while concurrently increasing the stability of phenolic compounds [2,4,10]. In order to remove fat, ground olive leaves were acidified with HCl to a pH value of 2.0 and were continuous extracted for 1 h with *n*-hexane at a ratio of 5:1 (v/w) in an orbital shaker (Orbital Shaker SO1, Stuart Scientific, Stone, Staffordshire, UK), at ambient temperature. The lipid extract was filtrated using GF/F filter paper in a Buchner funnel and the filtrate was removed. In order to investigate the extraction kinetics of antioxidants defatted residues of milled olive leaves were extracted using different extracting solvents (methanol, ethanol, mixture of ethanol:water 1:1, *n*-propanol, isopropanol and ethyl acetate), at solvent volume to sample mass ratio of 5:1 v/w, extraction times from 30 min to 24 h in an orbital shaker and at ambient temperature (25 °C). The sample mass ratio of 5:1 was derived through experimentation involving the effect of liquid to solid ratio. This step was investigated using solvent volume to sample mass ratios ranging from 2:1 to 7:1 v/w, ambient temperature and extraction time of 3 h. The extracts were filtrated using GF/F filter paper in a Buchner funnel to obtain the filtrate. The filtrates were evaporated to dryness in a rotary evaporator (Ika-Werke RV06-ML, Staufen, Germany) and the residue redissolved in methanol and kept at −20 °C until subsequent analyses. At least three extraction replicates were made for each condition tested.

#### 2.2.2. Supercritical Fluid Extraction

SFE extractions were performed using samples of 2 g at 40 °C, 350 bar and for 60 min and a Supercritical Fluid Extraction System (SFX System 5100, ISCO Inc., Lincoln, NE, USA), which consists of an extractor (SFX 3560) and two syringe pumps Model 100DX as previously described [4].

### 2.3. Phenolic Content Determination

The total phenol content of olive leaves extracts was determined colorimetrically, using the Folin-Ciocalteau reagent according to Lafka *et al.* [10] which is a modification of Gutfinger’s [14] method. Measurements were performed at 725 nm by using a double-beam UV-VIS spectrophotometer Hitachi U-3210 (Hitachi, Ltd., Tokyo, Japan). Caffeic acid served as standard for preparing the calibration curve ranging from 60 to 140 μg/25 ml assay solution.

### 2.4. Antioxidant Activity

#### 2.4.1. DPPH Radical Scavenging Method

The antioxidant activity of phenolic extracts was evaluated by using the stable 2,2-diphenyl-1-picryl-hydrazyl radical (DPPH) according to the method of Bandoniene *et al.* [15] following a modification by Lafka *et al.* [10] and using a double-beam UV-VIS spectrophotometer Hitachi U-3210 (Hitachi, Ltd., Tokyo, Japan). The radical scavenging activities of the tested samples, was expressed as percentage inhibition of DPPH.

#### 2.4.2. Rancimat Method

Ethanol extracts of olive leaves were freeze-dried (Virtis 5L, Gardiner, NY, USA) and the freeze-dried extracts were added into commercial sunflower oil without any added antioxidant at concentrations ranging from 40 to 240 ppm. The antioxidant potential of these extracts was investigated and compared to the antioxidant potential of samples of commercial sunflower oil containing synthetic (BHT, ascorbyl palmitate) and natural (vitamin E) antioxidants. The measurements were performed in a Rancimat 679 Instrument (Metrohm, Herisau, Switzerland) with air flow-rate and temperature set at 20 l/h and 100 °C, respectively.

#### 2.4.3. Peroxide Value Determination

All solvent and SFE extracts were added at different concentrations (100 and 150 ppm) to commercial virgin olive oil and sunflower oil. Then, all the samples were put in an oven at 85 °C where thermal oxidation took place. Every 24 h the samples were analyzed for peroxide value in order to monitor the oxidation process. The peroxide value was determined according to the EEC [16] method. The peroxide value was expressed as mM of active oxygen per kg of sample.

### 2.5. Statistical Analysis

Data were analyzed with one-way ANOVA Post Hoc Tests and pairwise multiple comparisons were conducted with the Tukey’s honestly significant difference test. Model fitting and analysis were performed using Statistica software (Statistica Release 7, Statsoft Inc., Tulsa, OK, USA).

## 3. Results and Discussion

### 3.1. Composition and Phenol Determination

In Table 1, data on chemical analysis of wild olive leaves and especially, the total phenol content of olive leaves extracts are shown. The phenol content of extracts varied in response to different materials and solvents used (Table 1). Solvents could significantly (*p* < 0.05) affect total phenolics due to differences in solvent polarities, which might influence the solubility of various constituents present in olive leaves. Hence, the selection of the appropriate solvent is one of the most relevant steps in optimizing the recovery of plant phenolics. SFE olive leaves extract exhibited similar (*p* > 0.05) phenol content with *n*-propanol, isopropanol and ethyl acetate extracts (Table 1).

**Table 1:** Olive leaves characterization and total phenol content.

Parameter	Value ± SD
*Leaves composition (% w/w)*	
Moisture	50.5 ± 1.9
Total solids	49.5 ± 0.8
Ash	3.7 ± 0.9
Fat	1.2 ± 0.2
*Total phenols (% w/w)* ^1^	
Methanol extract ^2^	2.06 ± 0.18 a
Ethanol extract ^2^	2.73 ± 0.31 b
Ethanol:water 1:1 extract ^2^	2.48 ± 0.28 ab
*n*-Propanol extract ^2^	1.37 ± 0.14 c
Isopropanol extract ^2^	1.35 ± 0.19 c
Ethyl acetate extract ^2^	1.35 ± 0.12 c
SFE/CO_2_ extract	1.28 ± 0.22 c

^1^ Total phenols dry weight, expressed as caffeic acid equivalents; ^2^ Extraction time 3 h; Means in the same column and parameters with unlike letters differ significantly.

### 3.2. Solvent Extraction Kinetics

During the solid-liquid extraction process, an enrichment of the liquid phase in phenolic substances occurs through a complex mechanism. Many of the mathematical models proposed in the past to describe different substances extraction from vegetable materials are simply empirical equations that fit to experimental data, while some of them are based on mass transfer theory. Hence, we attempted to find which one of the most frequently used, namely, Peleg [17], second order, Elovich and the power law model, could best describe as well as better quantify the extraction rate of phenolics from wild olive leaves.

Peleg’s model, which actually is a hyperbolic model, was used in the form:

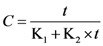
(1)
with K_1_ and K_2_ constants, *C* concentration and *t* extraction time. Constant K_1_ is related to the extraction rate at the beginning of the process (*t* = 0), while constant K_2_ is related to maximum extraction yield (*t → ∞*, phenolics concentration at equilibrium).

The second-order model is described by the equation:

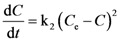
(2)
where k_2_ is the second-order extraction rate constant and *C*_e_ concentration at equilibrium. By integration Equation (2) gives:

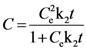
(3)
which can be rearranged as:

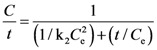
(4)
and finally as:

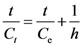
(5)
with 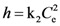 expressing the initial extraction rate *h*, and *C*_e_ equilibrium concentration (extraction capacity). 

The Elovich model can generally be expressed as:

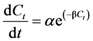
(6)
which can be rearranged as:

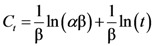
(7)
where *α* initial extraction rate and β extraction rate constant. Elovich’s equation is a logarithmic relationship, which is derived by assuming that the extraction rate decreases exponentially with increasing extraction yield.

The power law model is used to reveal the diffusion mechanism of an active substance through non-swelling particles and is described by:

*C* = B*t^n^* (8)

where B is a constant incorporating the characteristics of the particle-active substance system and *n* is the diffusion exponent, characterizing the mass transfer mechanism. In most vegetable materials, its values are lower than 1.

**Figure 1:**
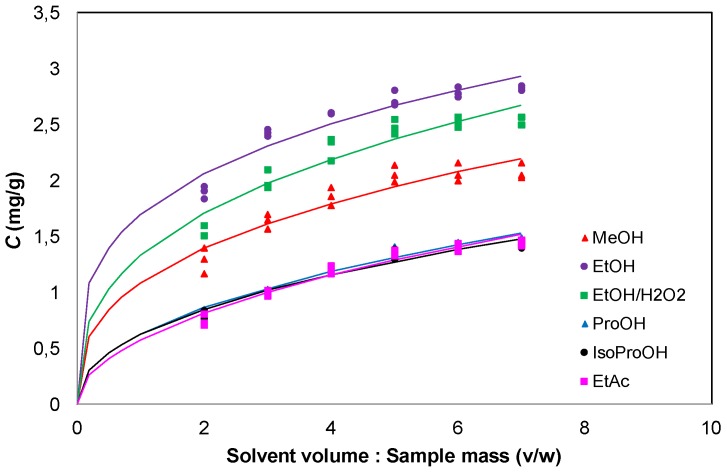
Effect of solvent/sample ratio (v/w) on the quantity of extracted phenols from olive leaves.

Solvent and solvent to material ratio affected the amount of phenolics extracted (Figure 1). High solvent/material ratios led in larger concentration gradients during diffusion from material interior into solution, and hence the extraction efficiency was increased (Figure 1). However, higher solvent/material ratios mean more solvent usage during extraction. Consequently, it is essential to recommend a suitable solvent/material ratio, which could be the beginning of a plateau, though a real plateau does not exist. Hence, a solvent/sample ratio of 5:1 (v/w) was the most suitable for the maximum extraction of phenolic compounds regardless of the extraction solvent. The most efficient solvent was ethanol, followed by ethanol:water 1:1 and methanol, while no differences (*p* > 0.5) were found among the other solvents.

**Figure 2:**
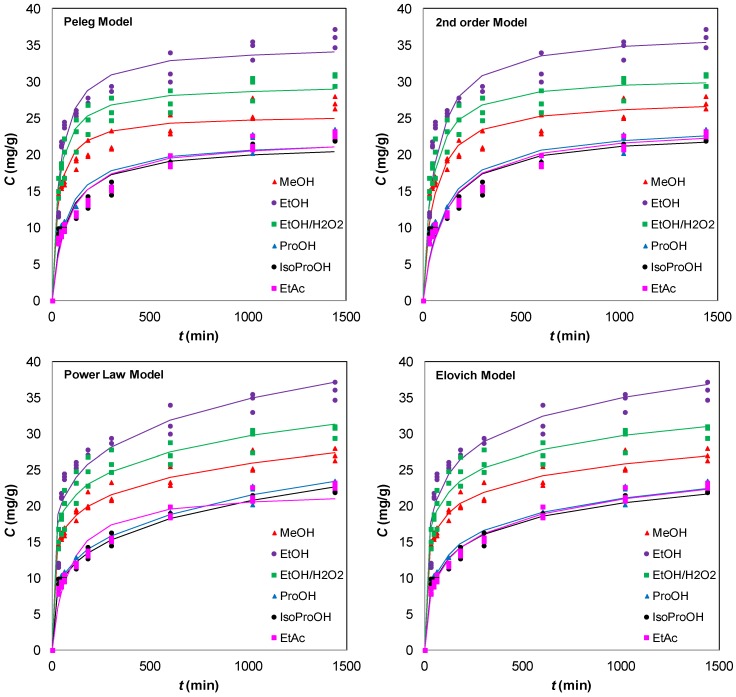
Kinetics of phenolics extraction from olive leaves with different solvents at 25 °C.

Figure 2 shows model fitting, while Table 2 presents constants for the models applied. It is evident that all of the models used described the experimental data with a good correlation. It should be noted that because they are empirical models, it is difficult to assign a physical meaning to their parameters. The second order kinetic model provided a best-fit description for the extraction of olive leaf phenolics for all solvents used. The initial leaching rate, *h*, appeared to be solvent-dependent, exhibiting values of 0.519–0.697 for methanol, ethanol and ethanol:water 1:1, while its values dropped to 0.218–0.229 for *n*-propanol, isopropanol and ethyl acetate. Moreover, the extraction capacity in the ethanol and ethanol to water 1:1 was higher than those for other solvents. The Elovich model provides a description of solid-liquid extraction of olive leaves phenolics that is better than that offered by the Peleg’s and power law models but not as good as that given by the second-order model. As can be seen from Table 2, both the second order and Elovich models could be considered the most appropriate due to their high correlation coefficients relative to other kinetic models. It is possible to observe that both the initial extraction rate *α* and the extraction rate constant β are solvent-dependent (Table 2). The experimental results could also be represented by Peleg’s and power law models, which showed correlation coefficients higher than 0.945 and up to 0.996 depending on extraction solvent (Table 2). Figure 2 demonstrates a good agreement of the experimental data using Peleg’s model for solid-liquid extraction of total phenols from olive leaves. The power law model, suitable for the diffusion of an active agent such as phenolics, gave a diffusion exponent *n* < 0.5 for all solvents used showing that Fickian diffusion predominated during phenolics extraction from olive leaves.

**Table 2:** Application of extraction models to phenolics extraction from olive leaves using various solvents.

Model	Methanol		Ethanol		Ethanol:Water 1:1		*n*-Propanol		Isopropanol		Ethyl Acetate	
	Parameter Values	*R*^2^	Parameter Values	*R*^2^	Parameter Values	*R*^2^	Parameter Values	*R*^2^	Parameter Values	*R*^2^	Parameter Values	*R*^2^
*Peleg*												
K_1_	1.159 ± 0.116	0.959	1.114 ± 0.102	0.967	1.034 ± 0.089	0.971	3.233 ± 0.368	0.945	3.424 ± 0.376	0.949	3.734 ± 0.355	0.963
K_2_	0.039 ± 0.001		0.029 ± 0.001		0.034 ± 0.001		0.045 ± 0.002		0.047 ± 0.002		0.045 ± 0.001	
*Second-order*												
k_2_	0.0007 ± 0.0001	0.995	0.0005 ± 0.0001	0.996	0.0007 ± 0.0001	0.996	0.0004 ± 0.00005	0.990	0.0004 ± 0.00004	0.993	0.0004 ± 0.00004	0.992
*h*	0.519 ± 0.089		0.644 ± 0.093		0.697 ± 0.128		0.229 ± 0.027		0.225 ± 0.023		0.218 ± 0.022	
*C*_e_	27.535 ± 0.394		36.731 ± 0.478		30.807 ± 0.393		24.215 ± 0.482		23.313 ± 0.386		23.935 ± 0.421	
*Elovich*												
*α*	10.043 ± 2.363	0.979	5.438 ± 1.880	0.935	11.501 ± 4.545	0.943	1.079 ± 0.144	0.980	1.028 ± 0.134	0.980	0.850 ± 0.078	0.980
β	0.312 ± 0.013		0.200 ± 0.015		0.271 ± 0.019		0.269 ± 0.011		0.277 ± 0.011		0.258 ± 0.008	
*Power law*												
B	8.935 ± 0.356	0.988	10.269 ± 0.996	0.945	10.541 ± 0.766	0.960	3.790 ± 0.158	0.994	3.649 ± 0.152	0.994	3.367 ± 0.119	0.996
*n*	0.154 ± 0.007		0.177 ± 0.016		0.150 ± 0.012		0.251 ± 0.007		0.251 ± 0.007		0.266 ± 0.006	

In all cases, the equilibrium concentrations were reached after 180 min (Figure 2). However, it should be noted that, depending on plant material, the equilibrium concentration could be reached even less than 3 h and extraction times lower than 1 h have also been reported [18,19]. The fast extraction process could be attributed to the fact that only those phenolic compounds more weakly linked to cell walls and those contained in vacuoles can presumably be easily recovered [18].

In olive leaves, the use of ethanol resulted in maximum recovery of phenolic compounds (Table 1 and Figure 2). Moreover, extraction with a mixture of ethanol to water 1:1 led to similar phenolic yield (*p* > 0.05) with ethanol extract (Table 1 and Figure 2). Methanol gave extracts with high phenol content and its phenolic yield did not differ significantly (*p* > 0.05) with the yield of ethanol/water extract, but differed significantly (*p* < 0.05) with the yield of ethanol extract (Table 1). Although high yield was achieved using methanol for the extraction of phenols from olive leaves, methanol is not a food grade solvent due to its high toxicity. *n*-Propanol gave similar phenolic yields (*p* > 0.05) to isopropanol and to ethyl acetate in olive leaves (Table 1 and Figure 2). Yields of 90.8% and 75.5% were achieved by the extraction of phenolic compounds with mixture of ethanol to water 1:1 and methanol respectively, comparing to extraction with ethanol. Extraction with *n*-propanol gave a yield of 50.2% and a yield of 49.5% was obtained using isopropanol and ethyl acetate as extracting solvents. As a consequence, ethanol was selected as the most appropriate solvent for the extraction of phenolic compounds from olive leaves for production of extracts with high phenol content and high antioxidant activity.

### 3.3. Antioxidant Activity of Phenolic Extracts

Ethanol extracts exhibited the highest antiradical activity, followed by methanol extracts (Table 3). On the other hand, the *n*-propanol extract of olive leaves significantly (*p* < 0.05) exhibited the lowest antioxidant activity (Table 3). The radical scavenging activity of ethanol olive leaves extract was significantly (*p* < 0.05) higher than the antioxidant activity of the other solvent extracts (Table 3). SFE wild olive leaves extract exhibited similar (*p* > 0.05) antioxidant activity with isopropanol extract and significantly different (*p* < 0.05) from the other solvent extracts (Table 3). The differences could be attributed different solvents and solvent polarity. Although *n*-propanol and isopropanol gave similar phenolic yields, the *n*-propanol extract of olive leaves showed significantly (*p* < 0.05) lower antioxidant activity than the isopropanol extract (Table 3).

**Table 3:** Antioxidant activity of olive leaves extracts ^1^.

Olive Leaf Extract	Antioxidant Activity, as % Inhibition
Methanol extract	46.2 ± 0.75 a
Ethanol extract	55.0 ± 1.09 b
Ethanol/water 1:1 extract	40.9 ± 1.22 c
*n*-Propanol extract	18.8 ± 0.58 d
Isopropanol extract	32.8 ± 1.41 e
Ethyl acetate extract	22.5 ± 0.63 f
SFE/CO_2_ extract	33.9 ± 1.81 e

^1^ Extraction time 3 h; Means with unlike letters differ significantly (*p <* 0.05).

The induction periods of sunflower oil subjected to accelerated oxidation conditions without or with added ethanol extracts and/or BHT, ascorbyl palmitate and vitamin E, are reported in Table 4. The antioxidant potential decreased according to this sequence: ethanol olive leaves extract + ascorbyl palmitate > ethanol olive leaves extract > vitamin E > ascorbyl palmitate > BHT (Table 4). Ethanol extracts of olive leaves increased the induction time of sunflower oil from 7.45 h to 12.97 h. Vitamin E, ascorbyl palmitate and BHT were proven poor protectors against oil oxidation with induction times which did not differ significantly (*p* > 0.05) and lower than that of ethanol extract (Table 4). But, the combined use of ethanol extract with ascorbyl palmitate increased significantly (*p* < 0.05) the induction time of sunflower oil to 33.05 h for olive leaves. Synergistic actions between synthetic only, natural and synthetic and natural antioxidants have been observed (ascorbic acts as a metal complexing agent and ascorbyl palmitate as a lipophile). It should be emphasized that the antiradical activity depends on the extract concentration and increased antioxidant activity was found with increasing extract concentration. However, the concentration leading to maximum antioxidant activity is closely dependent on the extracts and, for the same extract, is dependent on the antioxidant activity test. The phenolic extracts of olive leaves acted as antioxidants in the narrow range of 50 to 200 ppm; outside of this range they acted as prooxidants. Generally, the antioxidants extracted from plants can show prooxidant activity at low concentration and antioxidant activity over certain critical values. Ethanol extracts exerted good protection against oxidation. The high antioxidant activity of ethanol extract can be attributed to components such as catechin, luteolin, caffeic acid and oleuropein [20,21,22]. As regards the antioxidant activity of oleuropein, which is present in high content in olive leaves extracts, is mainly due to the hydroxytyrosol moiety in its structure [23]. In addition, the hydroxylated cinnamic acids are more effective than their benzoic acid counterparts and activity becomes marked in caffeic acid, while tyrosol exhibits lower antioxidant power.

**Table 4:** Induction period at 100 °C of sunflower oil without or with the addition of synthetic and natural antioxidant ^1^.

Sample	Induction Period ^1^ (h)	Protection Factor ^2^
Sunflower oil	7.45 ± 0.07 a	1.00
Sunflower oil + ascorbyl palmitate ^3^	9.97 ± 0.38 b	1.34
Sunflower oil + BHT ^3^	10.23 ± 0.13 b	1.37
Sunflower oil + vitamin E ^3^	9.20 ± 0.41 b	1.23
Sunflower oil + ethanol extract ^4^	12.97 ± 0.49 d	1.74
Sunflower oil + ethanol extract ^4^ + Ascorbyl palmitate ^3^	33.05 ± 0.45 g	4.44

^1^ Mean values ± SD; ^2^ Protection factor: induction period of sample/induction period of sunflower oil; ^3^ 200 ppm; ^4^ 150 ppm; Means in the same column with unlike letters differ significantly (*p <* 0.05).

**Figure 3:**
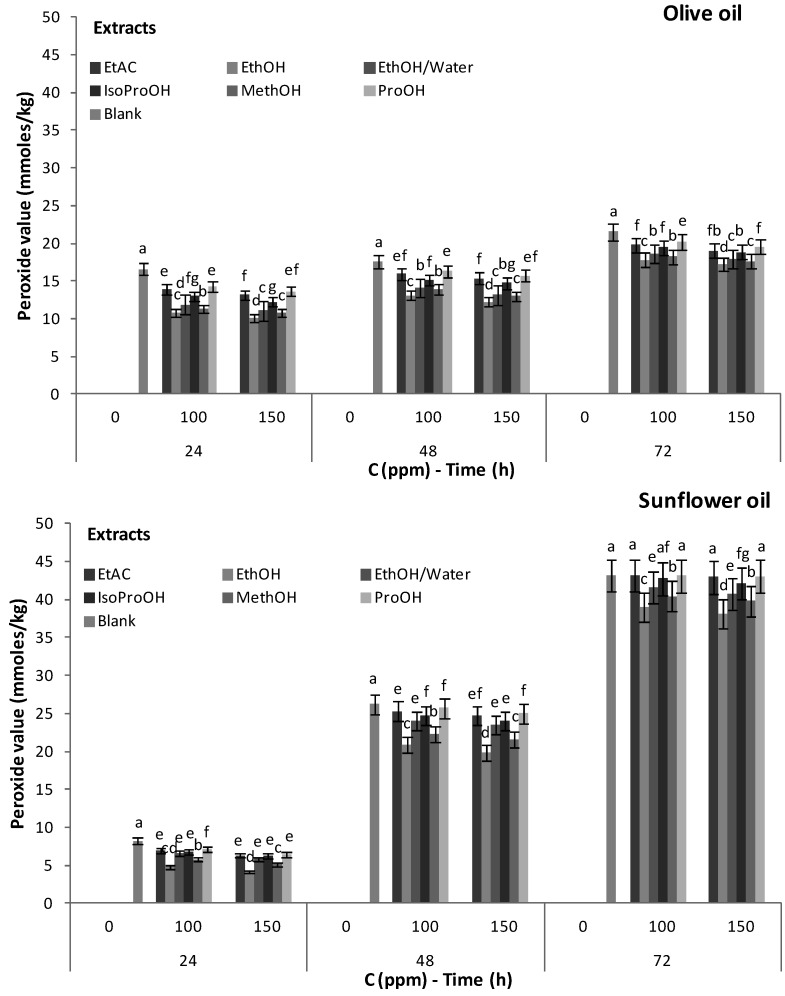
Peroxide value (mM/kg) of olive oil and sunflower oil enriched or not enriched with natural phenolic antioxidants from olive leaves (*T* = 85 °C).

The increase in heating time resulted in an increase of peroxide value in all samples (Figure 3). The antioxidant capacity of phenolic compounds was reduced because of thermal reduction of phenolic molecules and/or because they were used in the protection of oil against oxidation. The oxidation rate of olive oil and sunflower oil after phenolic extract addition, decreased significantly, even after three days (Figure 3). Furthermore, a 150 ppm phenolic extract addition concentration led to higher inhibition of oxidation than that of 100 ppm in both methanol and ethanol extracts (Figure 3). This was also valid for ethanol:water, propanol, iso-propanol and ethyl acetate extracts in olive oil. Ethanol extract exhibited the highest antiradical activity, followed by the methanol extract, while the *n*-propanol extract showed the lowest antioxidant activity. The above results are in agreement with those obtained by the DPPH method (Table 3 and Figure 3). The SFE/CO_2_ olive leaves extract showed similar (*p* > 0.05) antioxidant activity with the isopropanol extract in olive oil and with the ethanol/water, isopropanol and ethyl acetate extracts in sunflower oil (Figure 3). More specifically, in sunflower oil ethanol:water and ethylacetate extracts showed no statistically significant differences (Figure 3). Compared with the control sample, the formation of peroxides, after 24 h of heating, decreased to about 17.8%–38.5% and 21.5%–49.0% in olive oil and sunflower oil samples respectively, with added olive leaves phenolic extracts, at a concentration of 150 ppm.

## 4. Conclusions

The second order kinetic model was proven as the best in correlating the experimental data, followed by the Elovich model. The nature of the solvent affects the mechanism of extraction. Ethanol was proven and selected as the most appropriate solvent for the extraction of phenolic compounds from olive leaves for production of extracts with high phenol content and high antioxidant activity. For constant temperature, at pH 2.0 and solvent to sample ratio 5:1 v/w the optimum time was 180 min. CO_2_-SFE of wild olive leaves led to recovery of phenolic compounds with relatively high antioxidant activity, compared to some of the solvents used. Olive leaves could be a low-cost, renewable and abundant source of phenolic antioxidants, with potent use in fatty foods.
